# Practical handling of allergic reactions to COVID-19 vaccines: A position paper from German and Austrian Allergy Societies AeDA, DGAKI, GPA and ÖGAI

**DOI:** 10.1007/s40629-021-00165-7

**Published:** 2021-04-19

**Authors:** Ludger Klimek, Karl-Christian Bergmann, Randolf Brehler, Wolfgang Pfützner, Torsten Zuberbier, Karin Hartmann, Thilo Jakob, Natalija Novak, Johannes Ring, Hans Merk, Eckard Hamelmann, Tobias Ankermann, Sebastian Schmidt, Eva Untersmayr, Wolfram Hötzenecker, Erika Jensen-Jarolim, Knut Brockow, Vera Mahler, Margitta Worm

**Affiliations:** 1 Center for Rhinology and Allergology, Wiesbaden, Germany; 2 Clinic for Dermatology, Venereology and Allergy, Charité—University Medicine Berlin, Charitéplatz 1, 10117 Berlin, Germany; 3 Berlin Institute of Health, Charité—Medical University Berlin, Berlin, Germany; 4 Outpatient Clinic for Allergology, Occupational Dermatology and Environmental Medicine, General Dermatology and Venereology, Department of Skin Diseases, Münster University Hospital, Münster, Germany; 5 Department of Dermatology and Allergology, University Hospital Marburg, UKGM, Philipps University Marburg, Marburg, Germany; 6 Department of Dermatology and Allergology, University Hospital Basel, University of Basel, Basel, Switzerland; 7 Department of Dermatology and Allergology, Giessen University Hospital, UKGM, Justus Liebig University Giessen, Giessen, Germany; 8 Clinic and Polyclinic for Dermatology and Allergology, University Hospital Bonn, Bonn, Germany; 9 Clinic and Polyclinic for Dermatology and Allergology at Biederstein, Technical University of Munich, Munich, Germany; 10 Department of Dermatology and Allergology, RWTH Aachen University Hospital, Aachen, Germany; 11 Pediatric and Adolescent Medicine, Bethel Children’s Center, OWL University Hospital, Bielefeld University, Bielefeld, Germany; 12 Clinic for Pediatric and Adolescent Medicine, Municipal Hospital Kiel GmbH, Kiel, Germany; 13 Center for Pediatric and Adolescent Medicine, Clinic and Polyclinic for Pediatric and Adolescent Medicine, University Medicine Greifswald, Greifswald, Germany; 14 Institute for Pathophysiology and Allergy Research, Center for Pathophysiology, Infectiology and Immunology, Medical University of Vienna, Vienna, Austria; 15 Clinic for Dermatology and Venereology, Allergy Center, Kepler University Hospital GmbH, Linz, Austria; 16 Institute for Pathophysiology and Allergy Research, Center for Pathophysiology, Infectiology and Immunology, Medical University of Vienna, Vienna, Austria; 17 Inter-university Messerli Research Institute Vienna, Vienna, Austria; 18 Paul-Ehrlich-Institute, Langen, Germany; 19 Allergology and Immunology, Department of Dermatology, Venereology and Allergology, Charité—University Medicine Berlin, Berlin, Germany

**Keywords:** Corona virus, Vaccination, Allergic reaction, Anaphylactic reaction, Safety

## Abstract

**Background:**

For the preventive treatment of the 2019 coronavirus disease (COVID-19) an unprecedented global research effort studied the safety and efficacy of new vaccine platforms that have not been previously used in humans. Less than one year after the discovery of the severe acute respiratory syndrome coronavirus 2 (SARS-CoV-2) viral sequence, these vaccines were approved for use in the European Union (EU) as well as in numerous other countries and mass vaccination efforts began. The so far in the EU approved mRNA vaccines BNT162b2 and mRNA-1273 are based on similar lipid-based nanoparticle carrier technologies; however, the lipid components differ. Severe allergic reactions and anaphylaxis after COVID-19 vaccination are very rare adverse events but have drawn attention due to potentially lethal outcomes and have triggered a high degree of uncertainty.

**Methods:**

Current knowledge on anaphylactic reactions to vaccines and specifically the new mRNA COVID-19 vaccines was compiled using a literature search in Medline, PubMed, as well as the national and international study and guideline registries, the Cochrane Library, and the Internet, with special reference to official websites of the World Health Organization (WHO), US Centers for Disease Control and Prevention (CDC), Robert Koch Institute (RKI), and Paul Ehrlich Institute (PEI).

**Results:**

Based on the international literature and previous experience, recommendations for prophylaxis, diagnosis and therapy of these allergic reactions are given by a panel of experts.

**Conclusion:**

Allergy testing is not necessary for the vast majority of allergic patients prior to COVID-19 vaccination with currently licensed vaccines. In case of allergic/anaphylactic reactions after vaccination, allergy workup is recommended, as it is for a small potential risk population prior to the first vaccination. Evaluation and approval of diagnostic tests should be done for this purpose.

## Introduction

Vaccination is rightly considered the gold standard in prophylaxis of infectious diseases. Previous mass vaccination strategies have been successful and have led to the complete elimination of serious infectious diseases (including smallpox and polio).

In the severe acute respiratory syndrome coronavirus 2 (SARS-CoV-2) pandemic, research efforts to elucidate immunopathology and develop vaccines have been and continue to be conducted [1, 2].

In December 2020, BNT162b2 (BioNTech-Pfizer company, BionTech, SE, Mainz, Germany, trade name Comirnaty®), the first mRNA vaccine, was licensed in the United Kingdom (UK) for the prevention of 2019 coronavirus disease (COVID-19); shortly thereafter, approvals were also granted in the United States, Canada, the European Union (EU), and many other countries worldwide, as well as for mRNA-1273 (Moderna, Inc., Cambridge, MA, USA), another mRNA vaccine. Both vaccines are lipid nanoparticle-formulated, nucleoside-modified mRNA vaccines [3, 4].

mRNA vaccines use the messenger ribonucleic acid to encode the protein of interest, but protein biosynthesis occurs in the human host cell.

Allergic and also anaphylactic reactions to the active ingredients of a vaccine itself or to other components of the vaccine preparation are among the potential risks of any vaccine product [5, 6].

Anaphylactic reactions occurred shortly after the start of BNT162b2 and mRNA-1273 vaccinations in the United Kingdom (UK), Canada, and the United States [7–10].

Allergic reactions to vaccines in general, including severe anaphylaxis, can be IgE-mediated, but also IgG- and complement-mediated. Usually, anaphylaxis occurs within the first 30 min after vaccination [5, 6].

Symptoms include urticaria with generalized itching, erythema, angioedema, especially swelling of the tongue and larynx, asthmatic symptoms with wheezing, coughing and dyspnea, and also tachycardia, hypotension, dizziness and vomiting. In worst cases, anaphylactic reactions can be lethal. In connection with the administration of the COVID-19 vaccines approved in the EU, no deaths from anaphylactic reactions have been reported worldwide. Severe anaphylactic reactions to vaccines are very rare and the rate has been estimated at 1.31 (95% confidence interval [CI] 0.90–1.84) per million vaccine doses [5].

## *Morbidity and Mortality Weekly Report*

A *Morbidity and Mortality Weekly Report* from the US Centers for Disease Control and Prevention (CDC) describes allergic reactions including anaphylaxis after receiving the first dose of BNT162b2 vaccine in the US [11].

From December 14 to December 23, 2020, a total of 4393 (0.2%) adverse events were reported to the Vaccine Adverse Event Reporting System (VAERS) following administration of 1,893,360 initial doses of BNT162b2 vaccine. Among these, 175 case reports were identified for further review as possible cases of severe allergic reaction, including anaphylaxis, based on description of signs and symptoms. The report notes that 21 of these cases met the case definition criteria for anaphylaxis, representing an estimated rate of 11.1 cases per 1 million doses administered. The median interval from receipt of vaccine to onset of symptoms was 13 min (2–150 min). In most of the patients (71.4%), symptoms appeared within 15 min, in 14.3% within 15 to 30 min, and in 14.3% after 30 min [11].

In 19 of 21 (90%) cases, patients were treated with epinephrine, 4 patients (19%) required hospitalization (including three in an intensive care unit), and 17 (81%) were treated in an emergency department. No deaths from anaphylaxis were reported after receipt of BNT162b2.

According to the report, 17 (81%) of the 21 patients with anaphylaxis had a documented history of allergies or allergic reactions, including to medications or medical products, foods, and insect stings. In addition, 7 patients (33%) had a positive history of anaphylaxis in the past, including one after rabies vaccination and another after influenza A (H1N1) vaccination.

In addition, the report notes that cases occurred after receiving doses from multiple different vaccine batches. Furthermore, 83 cases of non-anaphylactic allergic reactions were documented after vaccination with BNT162b2—with symptom onset within one day, of which 72 (87%) were classified as non-severe. The most commonly reported symptoms included pruritus, rash, pharyngeal itching and irritation, and mild respiratory symptoms. The European Medicines Agency (EMA), CDC and US Food and Drug Administration (FDA) will continue to conduct increased surveillance for anaphylaxis in recipients of COVID-19 vaccine [11, 12].

## Potential anaphylaxis-inducing ingredients in mRNA-COVID-19 vaccines

The COVID-19 vaccines currently commercially available in Western industrialized countries, BNT162b2 and mRNA-1273, do not contain any of the “classical” allergy-inducing components such as gelatin, hen’s egg or cow’s milk proteins (mainly responsible for immediate type reactions), thiomersal (organic mercury compound), aluminum, phenoxyethanol, or formaldehyde (mainly responsible for late type reactions). Residues of antimicrobial substances such as neomycin or substances such as latex, yeasts and dextran are not included as far as known, and preservatives or other additives are not required [3, 4, 7, 9].

Therefore, it is first necessary to clarify which components of BNT162b2 and mRNA-1273 (Table 1) can generally induce anaphylaxis.

**Table 1:** Ingredients listed in BNT162b2 and mRNA-1273. (After [13, 14])

BNT162b2	mRNA-1273
Nucleoside-modified mRNA encoding the viral spike (S)-glycoprotein of SARS-CoV‑2	Nucleoside-modified mRNA encoding the viral spike (S)-glycoprotein of SARS-CoV‑2
2((polyethylene glycol)-2000)-N,N-ditetradecylacetamide	Polyethylene glycol (PEG) 2000 dimyristoyl glycerol (DMG)
1,2-distearoyl-sn-glycero-3-phosphocholine	1,2-distearoyl-sn-glycero-3-phosphocholine
Cholesterol	Cholesterol
((4-hydroxybutyl)azanediyl)bis(hexane‑6,1‑diyl)bis(2-hexyl decanoate)	SM-102 (patent from Moderna)
Potassium chloride	Tromethamine
Monobasic potassium phosphate	Tromethamine hydrochloride
Sodium chloride	Acetic acid
Dibasic sodium phosphate-dihydrate	Sodium acetate
Sucrose	Sucrose

Both vaccines consist of nucleoside-modified mRNA encoding the viral spike (S) glycoprotein of SARS-CoV‑2. This SARS-CoV‑2 viral mRNA is packed in lipid nanoparticles (LNPs) enhancing the transport of the mRNA into human cells. The liposomal shell consists essentially of phospholipids (often modified by incorporated cholesterols), which enclose the RNA vaccine in the aqueous environment [15]. These auxiliary substances, known as lipid nanoparticles, which serve both as carriers and as stabilizers of the RNA, are partly PEGylated, this means covalently bound to polyethylene glycol (PEG) 2000, that among other things builds a steric barrier against premature degradation of the liposomes through the reticuloendothelial system [16] and therefore increase their stability and lifetime. The LNP also work as an immune boosting adjuvant.

In principle, these components can act individually or in combination as inducers of immunological hypersensitivity reactions (HR). For example, both single- and double-stranded RNA can stimulate the innate immune system, e.g., via the Toll-like receptors TLR 3 and TLR 7/8 and can lead to the excessive release of various immune-activating cytokines [17]. Furthermore, depending on their size, composition, structure and surface charge, liposomes can activate the innate, as well as the acquired immune system, and induce the building of antibodies against specific constituents [15, 18]. Subsequently, auxiliary substances are also capable of triggering HR [19]. PEG could play a special role in this context.

PEGylated LNPs are present in both vaccines and it has been described that anaphylactic reactions rarely can be triggered by PEG [7, 9, 20–25], although it is present in many medications and everyday products.

Also established are PEGylated pharmaceutical active substances, in which PEG and the active substances are chemically linked. Remarkably, PEG has also been used in preparations for allergen immunotherapy (AIT) [26]. For example, a previous study showed that 50% of patients receiving subcutaneous AIT with PEGylated allergen extracts for the treatment of allergies to ragweed pollen or bee venom developed antibodies directed against PEG. These were predominantly of the IgM type, although clinical relevance could not be established [26]. Patients, previously having contact with PEG, can have antibodies against PEG, and this could be a risk for anaphylactic reactions when vaccinated with a vaccine containing PEGylated molecules [7, 9, 21].

PEGs of various chain lengths are used in numerous everyday products such as toothpaste, tooth whitening, dental floss, shampoos, cosmetic products, cosmetic-dermatological fillers, vitamin preparations and lozenges as thickeners or solvents, softeners or moisturizers, and they have been used for decades as laxatives in preparation for colonoscopy (macrogol). Furthermore, they are found in a wide range of medicines such as antibiotics, analgesics, antiemetics, antiepileptics, antidepressants, anticoagulants, even in anti-allergic drugs such as glucocorticoid preparations and antihistamines, as well as in products used in the medical field such as disinfectants or ultrasound gels. An increasing number of biopharmaceuticals and biologics also contain PEGylated compounds [27, 28].

PEGs are hydrophilic polyether compounds that have numerous synonyms (e.g., macrogol). The molecular weight of PEGs varies from 300 to 35,000 g/mol and HR to PEGs of all molecular weights, with both immediate- and late-type reactions are described [20, 23, 24, 27, 28].

HR to PEGs in the sense of antibody-induced anaphylaxis can be triggered either by PEG-specific IgE or IgM/IgG antibodies [8]. IgE-mediated anaphylaxis has been described in several case reports and primarily demonstrated by positive prick test reactions and correspondingly positive provocation tests [23, 24, 29–32].

PEG-specific IgE antibodies have been detected in sera from patients with PEG-induced anaphylaxis, but IgE-mediated allergies do not appear to be solely responsible for reactions to PEG [23, 27–29, 33]. Utilization of basophil activation test (BAT) has also been described but is so far limited to specialized centers and not part of daily routine diagnostics [23, 27–29, 33, 34].

Causing anaphylactic HR by PEG-specific IgM/IgG antibodies [18] can also occur in the context of complement activation-related pseudoallergy (CARPA), which is mainly triggered by nanoparticle-based drugs (which are often PEGylated) [35]. Binding of anti-PEG IgG and IgM to liposomes with subsequent complement activation has been described [18, 35].

Independent of PEGylation, liposomes themselves have the potential to activate complement unspecifically, depending on their surface structure and surface-bound components as well as their charge. Important mediators here are the complement products C3a, C4a and C5a (anaphylatoxins) [18].

Potentially cross-reactive substances to PEG include polysorbate, poloxamers, PEG stearates, lauromacrogol, PEG stearyl/ketyl ether (Table 5). For polysorbate in particular, the US CDC has defined a warning for potential reactions [36]. The CDC considers an immediate-type allergic reaction of any severity to polysorbate a contraindication for the mRNA vaccines available to date. These patients should not be vaccinated with mRNA vaccines [37].

In addition, and in contrast to the BNT162b2 vaccine, mRNA-1273 contains tromethamine, also known as trometamol (molecular formula: C_4_H_11_NO_3_) [3]. Tromethamine is an organic amine used in various drugs for topical, enteral or parenteral administration [38, 39] and also in cosmetic products as an emulsifier. Contact sensitization as well as immediate type allergy to tromethamine have been described, as well as anaphylaxis when used as an excipient in iodinated X‑ray and gadolinium-based products [38–40].

Which substances actually are responsible for triggering the observed anaphylaxis has not been clarified, yet.

## Results from clinical trials of mRNA-COVID-19 vaccines

### BNT 162b2

As outlined previously, BNT162b2 is a lipid nanoparticle (LNP)-formulated, nucleoside-modified mRNA vaccine. While the LNPs help protect the mRNA from enzymatic degradation and ensure efficient cellular uptake, the N‑methylpseudouridine (m1Ψ) nucleoside modification attenuates immune sensitization and supports increased RNA translation in vivo. The vaccine encodes the SARS-CoV‑2 spike glycoprotein in full length [41].

The phase‑I trial showed good safety for BNT162b2 with mild to moderate local reactions (swelling and pain at the injection site) and mild systemic reactions (mostly fever in up to 17% of participants) [42].

The BNT162b2 phase-II/III trial started in July 2020 with originally planned 30,000 participants aged 18–85 years, but a protocol amendment then expanded the enrollment to 44,000 participants and lowered the age of the participants to 12 years [4, 43–45].

The primary endpoint of the phase-II/III trial assessed the occurrence of confirmed COVID-19 disease with onset at least 7 days after administration of the second dose in study participants [4].

In the cohort of participants without evidence of existing or prior SARS-CoV‑2 infection (*n* = 36,258), the primary endpoint occurred in 8 verum and 162 placebo patients, respectively, corresponding to a predefined efficacy of 95% (95% CI 90.3–97.6%) [4].

Severe COVID-19 disease occurred in 4 study participants after the second dose (1 in the verum group and 3 in the placebo group) and in 10 study participants after the first dose (1 in the verum group and 9 in the placebo group). Because of the small number of severe cases of COVID-19 disease, statistical significance for efficacy in preventing severe COVID-19 disease could not be determined, but there was a clear trend in favor of BNT162b2 (66.4%, 95% CI −124.8 to 96.3). For the safety analysis, local and systemic adverse events occurring within 7 days after receiving vaccine or placebo were evaluated by self-reports in an electronic diary. The most common adverse drug reactions were injection site reactions (84.1%), fatigue (62.9%), headache (55.1%), muscle pain (38.3%), chills (31.9%), joint pain (23.6%), and fever (14.2%) [4]. Injection site pain, the most common adverse local reaction, was resolved within 1 to 2 days. In terms of systemic adverse reactions, fatigue (3.8%) and headache (2.0%) were the most common [4].

A few cases of anaphylaxis have been reported in the UK following vaccination with BNT162b2. The British authorities (Medicines & Healthcare Products Regulatory Agency, MHRA) initially warned against vaccination of patients with a known anaphylactic reaction to a vaccine, a drug or food. Due to a lack of evidence, the MHRA withdrew this warning on December 30, 2020. The FDA, MHRA and EMA have included anaphylactic reaction monitoring in their pharmacovigilance plan.

### mRNA-1273

mRNA-1273 is a nucleotide-based vaccine candidate encoding a prefusion-stabilized form of the full-length SARS-CoV‑2 spike (S) protein. Due to the labile nature of the mRNA, it is encapsulated and delivered via a lipid nanoparticle carrier (LNP). After injection of the vaccine into the muscle, myocytes take up the LNP carrier and release the mRNA into the cytoplasm for translation into the S protein.

The clinical development program for mRNA-1273 consists of three studies: A phase‑I (NCT04283461), a phase-II (NCT04405076) and a phase-III (NCT04470427) study.

The phase‑I clinical trial of mRNA-1273 began on March 16, 2020, and study results have been published [46].

A phase-II trial was initiated in May 2020 as a dose-finding study of mRNA-1273 50 µg or 100 µg versus placebo [47].

The phase-III study started in July 2020 and is available as an interim analysis. The final sample size is 30,000 participants.

The emergency approval of mRNA-1273 in the US and UK and the approval in the EU were based on early phase‑I and -II studies [48, 49] and on reviews of results from an ongoing phase-III study of 33,000 adult subjects randomized 1:1. The efficacy of two injections 28 days apart of 100 µg each of mRNA-1273 vaccine was compared against placebo.

The evaluation of the phase-III trial showed that the vaccine was 94.1% effective in preventing SARS-CoV‑2 infection 14 days after administration of the second dose. For the efficacy analysis, 196 cases were evaluated, of which 185 cases of COVID-19 were observed in the placebo group versus 11 cases in the mRNA-1273 group. The secondary endpoint included the evaluation of severe cases of COVID-19 and included 30 individuals. All of these severe cases occurred in the placebo group and none occurred in the mRNA-1273-inoculated group [50].

The phase‑I safety results showed adverse systemic events such as arthralgia, fatigue, fever, chills, headache, myalgia, nausea mild to moderate in severity after the first dose. Local adverse events (redness/erythema, induration/swelling, injection site pain) were predominantly rated as mild to moderate after both the first and second doses. Fatigue, chills, headache, myalgias, and injection site pain were common adverse events in more than 50% of participants after both vaccine doses [3].

In the phase-III study, adverse events occurred more frequently after the second dose and the majority of reported events resolved rapidly. The most common events were injection site pain (2.7%) after the first dose and fatigue (9.7%), myalgia (8.9%), arthralgia (5.2%), headache (4.5%), pain (4.1%), and injection site erythema/redness (2.0%) after the second dose [48–50].

## Recognition of an allergic reaction to COVID-19 vaccines

Allergic reactions to COVID-19 vaccination can occur in the sense of anaphylactic reaction with symptoms on the skin, respiratory tract, gastrointestinal tract and cardiovascular system, which are divided into 4 severity levels [6].

The reaction may principally start with prodromal symptoms of itching, burning sensation in the palms, soles and genital area, metallic taste, anxiety, headache and disorientation. Urticaria, oral mucosal discomfort, dysphagia as well as throat swelling and bronchial constriction with dyspnea are then common. In severe cases, airway obstruction and cardiovascular involvement can lead to a lethal course.

Characteristically, symptoms develop suddenly and shortly after application of an allergen; early onset makes a severe reaction more likely than delayed onset [6]. With the BNT162B2 vaccine, ¾ of all allergic reactions occurred within 15 min after vaccination [11].

The diagnostic differentiation of anaphylaxis from fear/panic reactions with hyperventilation can be difficult. It is important to classify symptoms and, if anaphylaxis is suspected, to initiate adequate treatment immediately. Blood sampling to determine serum tryptase (compared with basal tryptase) 1–2 h after a reaction is helpful in confirming the diagnosis of anaphylaxis [6, 51].

Delayed reactions of varying severity can also manifest themselves with the symptoms described above after hours and are therefore not documented at the vaccination center during the monitoring phase. In addition, late-type reactions (T-cell-mediated rashes) can occur after days.

## Therapy of anaphylaxis to COVID-19 vaccines

Anaphylactic reactions require immediate treatment, and the administration of volume intravenously and adrenaline intramuscularly is essential in cases of rapid progression and involvement of multiple organs (Table 2; [6, 51]). Proper positioning of the patient (shock position) is also important. Blood pressure, pulse, and oxygen status must be adequately monitored, and the administration of oxygen is recommended [6, 51]. Antihistamines are particularly effective in urticaria, and glucocorticoids counteract a biphasic course [6], among other effects. Because of the possibility of a biphasic course, monitoring of the patient for 24 h is recommended, at least in severe reactions [6].

**Table 2:** Pharmacotherapy of anaphylaxis for children, adolescents and adults in ambulatory conditions. (After [6])

Active ingredient	Application path	<7.5 kg bw	7.5–25 (–30)^d^ kg bw	30–60 kg bw	>60 kg bw
Adrenalin	Intramuscular	50–600 g			
Adrenalin	Autoinjector i.m.	Not allowed	150 µg	300 µg	1–2 × 300 µg or 500 µg
Adrenalin	Inhalation nebulizer	2 ml^b^			
Adrenalin	Intravenous^a^	Titrating boli 0.01 mg/kg bw			
Dimetinden	Intravenous	1 ml^c^	1 ml/10 kg bw^c^ (max. 4 ml)	1 Amp = 4 ml^c^	1–2 Amp = 4–8 ml^c^ (1 ml/10 kg bw)
Prednisolone	Intravenous	50 mg	100 mg	250 mg	500–1000 mg
Salbutamol Terbutaline	Inhalative	2 puffs DA per spacer	2 puffs DA per spacer	2–4 puffs DA per spacer	2–4 puffs DA per spacer
Volume	Bolus (NaCl 0.9%)	20 ml/kg bw	20 ml/kg bw	10–20 ml/kg bw	10–20 ml/kg bw
Oxygen	Inhalative	2 to 10 l/min	5 to 12 l/min	5 to 12 l/min	5 to 12 l/min

*bw* body weightm, *DA* dose aerosol

^a^For intravenous administration, dilute 1 ml of a 1 mg/ml epinephrine solution to 100 ml NaCl 0.9% (final concentration 10 g/ml)

^b^For inhalation, the stock concentration is used (1 mg/ml)

^c^A (stock) concentration of 1 mg/ml (1 ml contains 1 mg dimetindene maleate)

^d^Different weight-based approvals for different auto-injectors

Exanthematic reactions are treated with topical glucocorticoids depending on the severity in case of mild course and small extension otherwise with systemic glucocorticoids. Antihistamines have limited efficacy against pruritus.

In order to guarantee this therapy, a minimum supply of pharmaceuticals (Table 2) and medical material (Table 3) is required, which must be available in every vaccination center. In addition, vaccination personnel must be trained in the recognition and treatment of severe allergic reactions.

**Table 3:** Material equipment for the treatment of anaphylactic reactions in outpatient facilities/vaccination centers. (According to [6])

Stethoscope
Blood pressure monitor
Pulse oximeter, possibly blood glucose meter
Tourniquet, indwelling venous cannulae (in different sizes), tips, infusion set, tape for fixation of cannulae
Oxygen and nebulizer set with oxygen mask *(different sizes)*
Resuscitation bag with masks *(different sizes)*
Suction device
Guedel tube if necessary
Volume (e.g., balanced full electrolyte solution)
Drugs for injection: epinephrine, glucocorticoid, H1 receptor antagonist
Short-acting β2-agonist e.g. salbutamol for inhalation *(preferably as inhalation solution for use via nebulizer set with mask, if necessary alternatively as metered dose inhaler with inhalation aid/spacer/mask, autohaler or similar products)*
Automated external defibrillator

## Prevention of severe allergic reactions to vaccinations

For BNT162b2, 11.1 cases of anaphylaxis occurred per 1 million doses [11], in the further course the number decreased to 4.7 cases of anaphylaxis to 1 million doses [37].

Other novel vaccine formulations, such as the recently licensed vector-based Ebola vaccine, have a significantly higher incidence of anaphylaxis [52, 53].

There are risk factors that may exacerbate allergic reactions which need to be considered during taking the medical history, such as previous anaphylaxis, uncontrolled asthma, mastocytosis, or other mast cell diseases [54–56].

In addition, medications such as beta-blockers, which are commonly prescribed for cardiovascular disease, may increase the severity of an anaphylactic reaction, and interfere with the treatment of anaphylaxis. Other known cofactors for triggering or exacerbating an anaphylactic reaction include nonsteroidal anti-inflammatory drugs (NSAIDs), exercise/sports, obesity or alcohol consumption [57, 58].

It is currently unknown whether these cofactors also promote a severe allergic reaction after vaccine administration. Furthermore, it is not known whether increased disease activity can promote adverse effects of vaccination. Therefore, we recommend that patients, e.g., during an asthma exacerbation or with severe atopic dermatitis, postpone vaccination to a time with stable disease conditions.

There is no association of the incidence of anaphylaxis to vaccines to age, sex, asthma, atopy status, or having had previous minor reactions to the same substance [59, 60].

In summary, it is important that every vaccination site and vaccinator are prepared to recognize and treat severe allergic reactions (Tables 2 and 3).

## Preventive measures to control allergic reactions to COVID-19 vaccines in populations at potential risk

To minimize anaphylactic HR to a Covid-19 vaccination, people at potential risk of such a reaction should be identified whenever possible. At the present time, however, neither approval studies nor data from the spontaneous registration registers indicate any signals for populations at risk who have an increased risk of anaphylactic side effects when the COVID-19 vaccines are administered, except for known allergies to an ingredient and an anaphylactic reaction to the first vaccine dose.

Nevertheless, based on general allergological experience, it may seem advisable to take special measures for certain groups of people (see below) (e.g. a prolonged follow-up period after vaccination instead of the 15-minute follow-up period required in the SmPC (summary of product characteristics) or to initiate a presentation at an allergy center). Predictive in vivo or in vitro tests to predict or exclude the risk of anaphylactic reactions in COVID 19 vaccination currently do not exist. A decision must be made depending on the individual risk profile, to which conditions a vaccination is possible.

## Contraindications and populations at potential risk

With regard to contraindications to COVID-19 vaccines and populations at potential risk, four patient groups can be distinguished (Table 4):

**Table 4:** Absolute (1) and potential (2, 3, 4) risk populations for developing allergic reactions to COVID-19 vaccines

1.	Patients with immediate-type allergy/anaphylaxis to one or more ingredients of the vaccine or to substances that are cross-reactive to them or patients with an anaphylactic reaction to the first dose of vaccine
2.	Patients with late-type allergy to one or more ingredients of the vaccine or to substances that are cross-reactive to them
3.	Individuals with previous anaphylaxis of unclear cause
4.	Patients with known mastocytosis or anaphylaxis to different drugs or other vaccines

### 1. Patients with immediate-type allergy/anaphylaxis to one or more ingredients of the vaccine or to substances that are cross-reactive to them or patients with an anaphylactic reaction to the first dose of vaccine

Ingredients of BNT162b2 and mRNA-1273 are listed in Table 1. Examples of PEG-containing drugs or cross-reactive substances can be found in Table 5.

**Table 5:** PEG-containing drugs and cross-reactive substances

PEG-containing drugs	PEG cross-reactive substances
Laxantia (e.g., Laxofalk®, Movicol®, Molaxole®)	Polysorbates (e.g. polysorbate 80, E4331)
PEG-liposomal drugs (e.g. Caelyx®, active ingredient doxorubicin)	Poloxamers (e.g. Pluronic®; Kolliphor®)
Aetoxysklerol® (lauromacrogol 400)	PEG stearates (e.g. Tagat®)
Various tablets and capsules	Lauromacrogol (e.g. Aetoxysklerol®, Anaesthesulf®)
PEGinterferon β‑1a (Plegridy®)	PEG stearyl/ketyl ether (e.g. Brij®)

*PEG* polyethylene glycol

### 2. Persons with late-type allergy to one or more ingredients of the vaccine or to substances cross-reactive to them

For persons with proven late-type allergy to one or more ingredients of the vaccine or to cross-reactive substances, there is only a low risk of a systemic reaction according to the literature. In the literature, there are single case reports of generalized skin reactions in contact sensitization to formaldehyde in vaccines [61]. In earlier days thiomersal was used more commonly in vaccines as a preservative, nevertheless systemic reactions after vaccination with such vaccines were rare and it was recommended to strictly avoid skin contact with the vaccines [62]. PEGs can cause contact eczema in the sense of a T-cell-mediated eczema reaction [30, 63]. The patch test with PEG 400 is established (the existing approved patch test substance is currently temporarily unavailable). Whether patients with sensitization to PEG also react to the PEGylated substances in COVID-19 vaccines has not been investigated, yet. There is also a discussion of potential cutaneous sensitization with the risk of subsequent IgE-mediated HR by epidermal up-take of low-molecular-weight PEGs by ointments, creams, and other skin-care products [23]. It is an open question whether these patients react to the PEGylated LNPs in COVID-19 vaccines. The other substances used in the vaccines are not available as test substances, and non-irritant concentrations have not been published. In case of a late-type allergy to the vaccines or components, the intradermal test with the vaccine or its source materials can be discussed, although this test is neither established nor is there any current experience with it. In this case, the intradermal test should be read after 24, 48 and 72 h.

### 3. Individuals with previous anaphylaxis of unclear cause

In individuals with previous anaphylaxis of unclear cause, PEGs as additives could have been the trigger. Therefore, a further clarification of the cause should be carried out before vaccination, in particular with regard to the ingredients of the vaccine or cross-reactive substances.

### 4. Patients with known mastocytosis or with previous anaphylaxis to different drugs

Patients with mastocytosis or with previous anaphylaxis to different drugs develop anaphylaxis more frequently [64]. Among patients with mastocytosis, anaphylaxis occurs mainly in adult patients and in patients with systemic mastocytosis, especially indolent systemic mastocytosis. Typical trigger factors are hymenoptera stings and various drugs (histamine relievers, muscle relaxants, opiates and contrast media). Anaphylaxis after vaccination has also been described in isolated cases [65–70]. However, all case reports to date have involved pediatric patients who, by their nature, receive vaccinations more frequently than adults. Initial case reports of adult vaccinees with known mastocytosis showed no allergic/anaphylactic side effects to the COVID-19 vaccines [71]. Particularly in the case of anaphylaxis due to previous vaccinations, drugs, including medical interventions such as colonoscopies, surgeries under general anesthesia, etc., PEGs as auxiliary substances can very rarely be the cause, whereby in the vast majority of cases the active substances themselves are the trigger. Also, in cosmetics and other products listed in Table 5, corresponding ingredients can be the cause in very rare cases.

## Consequences of risk assessment for COVID-19 vaccination

People belonging to risk group 1 should not receive vaccination with the respective vaccine. Shortly, further COVID-19 vaccines with presumably other ingredients will be available, so that in case of proven allergy, especially to PEG, it can be recommended to switch to such a vaccine in the future. Successful tolerance induction in patients with HR to PEGylated interferon‑α is described in the literature, but these patients showed late-type allergies and it was not differentiated whether the allergy was due to sensitization to PEG or IFN‑α [72]. Such a tolerance induction with the vaccine itself appears from various points of view not practicable, in particular in view of the current shortage of vaccines.

People belonging to risk groups 2 and 3 require further allergological consultation and tests.

Individuals belonging to risk group 4 (mastocytosis) are recommended to carry their emergency epinephrine auto-injector kit (recommended for every patient with mastocytosis) regularly to/after vaccination [68] and the follow-up period should be prolonged to 30 min.

These recommendations do not exclude people belonging to risk groups 2–4 from COVID-19 vaccination. However, allergological–diagnostic measures are recommended for people belonging to risk groups 1–3, and increased safety standards should be applied for risk groups 1–4 or other described measures are to be taken.

In contrast, individuals with allergy of any severity to other allergens such as pollen, dust mites, fungal spores, animal epithelia, foods, insect venoms, or to drugs and excipients that are not vaccine ingredients or are cross-reactive do not represent a principal risk population for anaphylaxis to COVID-19 vaccines. With respect to allergic disease patterns, individuals who have atopic disease such as rhinoconjunctivitis allergica, bronchial asthma, atopic eczema, allergic contact dermatitis or drug rashes, urticaria, angioedema, or polyposis nasi are not at increased risk.

**Fig. 1:**
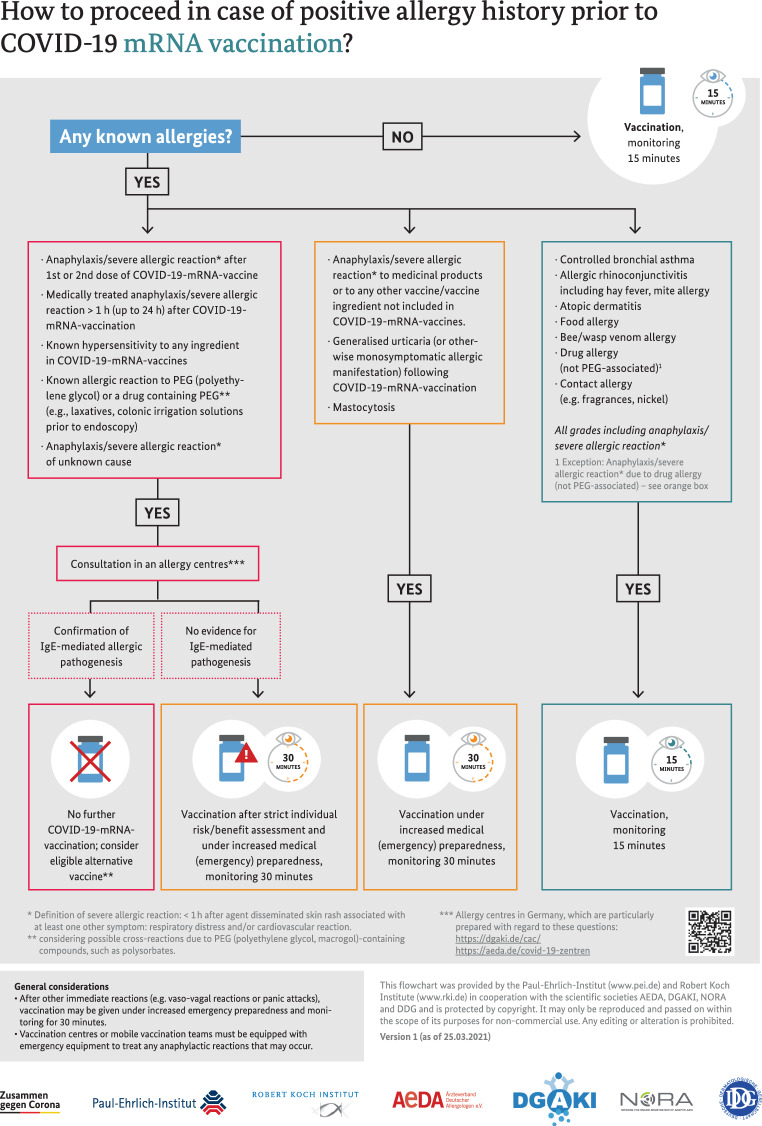
Flow chart for the procedure in case of different allergological diseases or anamnestic information. This flowchart was created by the Paul Ehrlich Institute (www.pei.de) and Robert Koch Institute (www.rki.de) in coordination with the specialist societies AeDA, DGAKI, working group on drug allergy of DGAKI, NORA and DDG and is protected by copyright. It may only be reproduced and passed on for non-commercial purposes within the scope of its purpose. Any editing or modification is not permitted. Version 1, dated February 26, 2021

A feasible traffic light scheme for patient identification is shown in Fig. 1, which was developed by the Paul Ehrlich Institute (www.pei.de) and Robert Koch Institute (www.rki.de) in coordination with the specialist societies AeDA (Aerzteverband Deutscher Allergologen), DGAKI (Deutsche Gesellschaft für Allergie und Klinische Immunologie), working group on drug allergy of DGAKI, NORA and DDG (Deutsche Dermatologische Gesellschaft).

## Diagnostic procedure for suspected allergic reactions to COVID-19 mRNA vaccines

The diagnostic procedure includes medical history, skin tests, challenge tests and laboratory tests.

### Medical history

The medical history should clarify a) the nature of the exposure to vaccine ingredients and b) identify clues to the form of the reaction mechanism of the HR experienced (Table 6). Reaction to different drugs (antibiotics, analgesics, antacids and laxatives, lozenges, cosmetic products as well as to depot glucocorticoids) should be considered as a possible indication for immediate-type sensitization to PEG [73] especially in reactions to different drugs with unrelated or potentially cross-reacting agents [73]. Overall, however, HR to PEG are rare.

- The following questions may be helpful in clarifying possible exposure to a vaccine ingredient:
  Did a severe allergic reaction occur in the setting of vaccination (<1 h after vaccination systemic rash, associated with at least one other symptom: shortness of breath and/or cardiovascular reaction)?
  Did a severe allergic reaction occur in the setting of a drug application (<1 h after drug intake systemic rash, associated with at least one other symptom: shortness of breath and/or cardiovascular reaction)?
  Is there a HR to an additive, e.g., PEGs? (Table 6)
- Of particular importance is the determination of whether the anamnestic HR represented an immediate or anaphylactic/anaphylactoid reaction.
  The following questions are indicative of this:
  Did the reaction occur within 30 min after exposure?
  Did an acute urticaria or angioedema occur?
  Did a rash persist for several weeks? (indicative for rather late-type reaction)
  Did systemic symptoms such as acute respiratory distress, vomiting, defecation/urination, circulatory symptoms such as dizziness, weakness, unconsciousness occur?
  Was epinephrine administered as part of the emergency treatment?

**Table 6:** Anamnestic questions with evidence of allergic reactions after PEG exposure

Questions with reference to allergies with PEG exposure
Did HR occur during colonoscopy/intake of laxatives?
Did HR occur as part of a surgical/invasive procedure?
Did HR occur after taking different medications or food products such as lozenges?
Did eczema/rashes occur after skin contact with cosmetics/care products/other possible PEG-containing products?

*HR* hyperreactivity reaction, *PEG* polyethylene glycol

Appropriate procedural protocols should be considered for anamnestic HR in the setting of invasive procedures.

### Skin tests

Skin tests can be performed if an immediate-type reaction to vaccine ingredients is suspected. These include prick and (in case of negative results) intradermal tests with the suspected substances, if available.

Currently, no approved test allergens are available for the named ingredients of the mRNA vaccines. Since it is not yet clear whether patients who are sensitized to PEGs also react to the PEGylated molecules, the diagnostic tests should be performed with the PEGylated molecules, but appropriate tests are not available, yet. It is important that PEGs of different molecular weights are used, since sensitization may differ depending on the molecular weight [74] and pure substances of pharmaceutical grade are used. According to literature, it is also recommended to exclude sensitization to polysorbate.

Since extreme rarely anaphylactic reactions have been observed in skin tests (prick and intradermal tests), highly sensitive patients should first be tested with diluted substance in a titrated approach (0.0001–100%) [24, 31].

Since reactions may also occur to substances used in the vaccination process but not contained in the vaccine, possible sensitization to latex, disinfectants and ethylene oxide should also be considered (ethylene oxide is a starting substance for the production of PEGs).

Adequate equipment and medication to intervention in the event of possible systemic HR must be kept on hand (Tables 2 and 3).

## In vitro laboratory diagnostics

A commercially available IgE assay for the ingredients of the vaccines is currently not available. Various experimental assays for the detection of IgE antibodies to PEGs have been described in the literature (including IgG and IgM), but their sensitivity and specificity have not been validated in larger collectives [23, 29]. However, corresponding validation studies are currently running. These assays should also ideally be performed with the PEGylated forms.

Positive basophil activation tests (BATs) for PEGs have been described [33], but also represent an experimental procedure that has not been extensively validated to date, and its use is reserved for experienced allergy centers [75].

### Laboratory diagnostics

should include determination of mast cell tryptase, as elevated levels ≥ 11.4 µg/ml may be indicative of mast cell activation syndrome or systemic mastocytosis, which carry an increased risk of more severe reactions in drug allergy [76, 77]. When clarifying HR in the context of medical procedures/interventions, it must also be remembered that latex (contained in syringes, gloves, etc.), disinfectants and ethylene oxide (sterilization of medical products; starting material of polymerization to PEGs) can also trigger immediate type reactions; these substances can also trigger allergic reactions in the context of vaccination.

### Provocation tests

In the case of negative or unclear results in skin tests and laboratory diagnostics, provocation testing can be carried out if necessary, if the suspicious products are available for this purpose, whereby testing of the individual substances should preferably be carried out first. Since severe HR are also possible, this testing is reserved for experienced allergy centers, taking into account the necessary requirements [76]. For example, oral provocation tests with PEG with an initial amount of 1 mg were performed, which was increased every 30 min up to a cumulative administration of 7.1 g, which corresponded to the minimum single dose of some laxatives, are described, although dedicated provocation protocols are not available [32]. In addition, the difference in the immunological compartments of an oral provocation test and an i.m. vaccination should be considered.

### In case of severe allergic reactions to the first COVID-19 vaccination,

we recommend clarification before the second vaccination. A decision on a case-by-case basis must be made about the indication for the second vaccination, taking into account the overall constellation and the test results. Alternatively, another vaccine can be used (as soon as available) that does not contain the putative allergenic components of the vaccine used in the first vaccination.

## Allergen immunotherapy

### Time delay between SCIT/SLIT and COVID-19 vaccinations

In principle, manufacturer-specific guidelines must be taken into account in the temporal relationship between allergen immunotherapy (AIT) and vaccinations. Thus, there should generally be an interval of about 1 week between SCIT (subcutaneous immunotherapy) and COVID-19 vaccination. Based on experience with other vaccinations, the following procedure has proven effective [78, 79]:

#### Initiation phase

If it is possible to perform the up-dosing phase of AIT (allergen immunotherapy) completely before the scheduled vaccination date, this can be done as usual and the recommendations given under “Maintenance therapy” then apply. If vaccination is imminent, initiation of SCIT or SLIT (sublingual immunotherapy) should be delayed until 1 week after the second vaccination date, if not otherwise recommended by the manufacturer.

#### Maintenance therapy

For the continuation of an ongoing AIT, we recommend a period of about 1 week between SCIT and vaccination, analogous to the above procedure, as well as at least 7 days interval after a vaccination, observing the minimum interval between 2 SCIT applications recommended by the manufacturer.

For SLIT, there are different recommendations from different manufacturers on the interval between vaccination and previous and subsequent SLIT administration. Therefore, no general recommendation can be given, but the information in the summary of product characteristics should be noticed and then an individual decision should be made. However, in order to be able to recognize possible side effects of SLIT or vaccination, we recommend suspending SLIT on the day of vaccination and to keep an interval of at least 3–7 days afterwards. In this case, SLIT can be taken up to the day before the vaccination.

## Biologics

### Procedure for therapy with biologics in connection with COVID-19 vaccination

The monoclonal antibodies mainly used in allergology are omalizumab, mepolizumab, benralizumab, reslizumab, dupilumab, and lanadelumab, whose indications and use in the SARS-CoV‑2 pandemic have already been pointed out in detail (Table 7; [80]):

**Table 7:** Evidence for COVID-19 vaccination in individuals with severe asthma, atopic dermatitis, urticaria, and chronic rhinosinusitis with nasal polyps who therefore are receiving biologic therapy

	Increased risk of infection	Live vaccines/attenuated live vaccines	Inactivated vaccines	Relevant additives
Omalizumab	Not known	No contraindication	No contraindication	Polysorbate 20
Mepolizumab	Decreased ability to fight parasitic infections in animal studies	No contraindication	No contraindication	Polysorbate 80
Benralizumab				Polysorbate 20
Reslizumab				
Dupilumab	Possible influence of parasitic diseases	*Contraindication*	No contraindication	Polysorbate 80
Lanadelumab	Not known	No contraindication	No contraindication	Polysorbate 80

*Anti-IgE (omalizumab)* is used particularly in severe allergic asthma, antihistamine-resistant chronic spontaneous urticaria, and chronic rhinosinusitis with polyposis nasi (CRSwNP).

*Antibodies for IL‑5 blockade (mepolizumab, benralizumab, reslizumab)* are used for severe eosinophilic/T2 asthma, and hypereosinophilia when appropriate.

*IL-4/13 blockade antibody (dupilumab)* is used for T2 asthma, atopic eczema, and chronic rhinosinusitis with polyposis nasi (CRSwNP).

The *plasma kallikrein inhibitor lanadelumab* is used for the treatment of hereditary complement-related angioedema.

In the current situation, it can be stated that adolescents and adults treated with omalizumab (Xolair®), mepolizumab (Nucala®), reslizumab (Cinqaero®), benralizumab (Fasenra®), dupilumab (Dupixent®) or lanadelumab (Takhzyro®) can in principle receive the COVID-19 vaccination without restriction with the currently approved vaccines.

With regard to the time interval between vaccination and therapy with the above-mentioned biologics, a distinction must be made between live vaccines and inactivated vaccines (dead vaccines). The previously approved COVID vaccines do not belong to either of the two previous vaccine classes, but form their own classes: the mRNA vaccines (BionTech and Moderna) and vector-based vaccines (AstraZeneca). However, since there are no recommendations for these vaccine classes so far, we assign them to the dead vaccines.

### No increased risk of infection or worse outcome from COVID-19 in asthmatics

It should be noted that individuals with bronchial asthma are not at increased risk for infection with SARS-CoV2 [80]. Also, individuals with bronchial asthma with mild to severe disease stages do not have an increased risk of having a more severe disease course in the event of COVID-19 disease [80]. Rather, the decisive factor for the course is whether they also suffer from a comorbidity; obesity, hypertension, and diabetes have the greatest negative impact [81]. A similar assumption can be made for vaccination, especially since the side effect reports of nearly 2 million patients vaccinated with BNT162B2 in the USA do not give a different signal for this [11].

It can also be established that patients taking biologics tolerate other protective vaccinations, such as influenza, like “normal persons”, i.e., no increased frequencies of side effects are known.

Similarly, there is no evidence for interactions of biologics and COVID-19 vaccination in individuals with atopic dermatitis, chronic idiopathic urticaria, or chronic rhinosinusitis with nasal polyps [82].

The fear that therapy with one of the five biologics could pose an additional risk in the case of vaccination or that the effect of the biologic could be cancelled out by the vaccine, or vice versa, therefore appears to be unfounded.

In the randomized controlled trials of omalizumab, the longest marketed biologic for individuals with severe allergic asthma [83] or chronic idiopathic urticaria [84], no exclusion criteria were defined for patients who recently had been or needed to be vaccinated during the trials. Also, no protocol is known from studies of the other biologics for use in severe asthma that examined the impact of vaccination. To date, there is also no known evidence or published data from routine care that would indicate that the effect of allergy-relevant biologics is altered by vaccination.

However, studies on the interaction of biologicals with vaccines should be carried out with regard to the allergological potential as well as the immunological efficiency of the vaccinations.

### Interval of 7 days between allergologically relevant biologics and vaccination is reasonable

There are no official recommendations regarding a time interval between the use of the biologics discussed here and the administration of vaccinations with inactivated vaccines in general. Since all vaccinations as well as the administration of biologics can potentially cause side effects, it makes sense for practical reasons to maintain a time interval. This allows a better estimation of a possible causal relationship between adverse reactions to vaccination and possible side effects of a biologic [85]. We therefore recommend an interval of approximately 7 days between the injection of a biologic and vaccination with mRNA-vaccines or vector-based vaccines or vice versa.

## Conclusion and key messages

The first approved vaccines against SARS-CoV‑2 in the European Union (EU) include BNT162B2, mRNA-1273 and, AstraZeneca AZD1222 (recombinant adenovirus ChAdOx1-S).

Shortly after approval, severe allergic reactions (anaphylaxis) to the mRNA-based vaccines were reported in single patients. The regulatory authorities of the EU, the USA and the UK agree that there is only an absolute contraindication to vaccination if there is an allergy to one of the vaccine components or if there was a severe allergic reaction to the first dose of the vaccine. It is important to mention that (as with any other vaccination) anaphylaxis can occur after vaccination, even if there is no history of allergic disease. Therefore, vaccination centers and other vaccinators should be prepared for prevention, diagnosis, and treatment of severe allergic reactions, and necessary medications and equipment should be available for immediate use in vaccination centers, as well as in care homes and for mobile vaccination teams. The presented potentially allergenic/immunogenic components should be tested in the above-mentioned risk population (risk groups 1–3) before vaccination, but also in patients after corresponding vaccination reactions, in order to identify the responsible allergen and to be able to take the necessary measures for the second vaccination dose if necessary. Alternatively, it may be possible to switch to another vaccine with a different composition that does not contain the potentially allergenic/immunogenic components. Testing with the vaccines themselves or its starting materials is recommended, but not always feasible due to the current shortage of materials. It is important to emphasize that currently there are no validated and approved test allergens and also no validated laboratory tests available for this indication. These should be made available whenever possible. It should also be pointed out that in particular pseudoallergic reactions cannot be detected by the test procedures described here and thus no comprehensive clarification of possible reactions to the vaccines is currently possible. Thus, no absolute safety for these patients with regard to possible allergic/pseudoallergic reactions is guaranteed despite testing.

In patients with mastocytosis, but also in patients with unclear minor allergic reactions of unknown cause in the anamnesis or after the first vaccination, premedication with antihistamines and possibly glucocorticosteroids may be useful before the (second) vaccination. Only patients with a high level of suspicion of a severe allergic reaction to an ingredient of the COVID-19 vaccine or substances that cross-react with them are currently not allowed to be vaccinated but may be able to switch to another vaccine preparation.

The authors emphasize that all information provided here reflects the current state of knowledge and should be continuously updated. Rapidly advancing knowledge may make it necessary to revise these recommendations in a short time.

### Key messages

- No allergy sufferer should be excluded from COVID-19 vaccination without sufficient reason.
- Except for a very small proportion of allergic persons with the defined contraindications or from the defined risk groups, allergic persons can receive COVID-19 vaccination with the currently approved vaccines.
- Persons belonging to the risk groups 1–3 (see above) should undergo an allergological evaluation prior to COVID-19 vaccination.
- For allergy sufferers who do not belong to the defined risk groups, there is no evidence for an increased risk from COVID-19 vaccination.
- Patients with allergies or who belong to a defined risk group (see above) should be monitored for 30 min after vaccination.
- However, anaphylactic reactions can occur even without known allergies. Every vaccinator must therefore be prepared for anaphylaxis treatments and have the appropriate expertise.
- Vaccinator and vaccination staff must be trained in the recognition and treatment of severe allergic reactions.
- For adequate treatment of possible anaphylaxis occurring in the course of vaccination, a minimum equipment of drugs and instruments (Table 3) must be available for immediate use in every vaccinating site (e.g., vaccination centers, physicians’ offices, mobile vaccination teams in nursing homes, patients’ residences).
- After (supposed) allergic reactions to the vaccines, an allergological work-up should be performed in a specialized center.
